# Nest Relocation and Excavation in the Florida Harvester Ant, *Pogonomyrmex badius*

**DOI:** 10.1371/journal.pone.0112981

**Published:** 2014-11-19

**Authors:** Walter R. Tschinkel

**Affiliations:** Department of Biological Science, Florida State University, Tallahassee, Florida, United States of America; Universidade de São Paulo, Faculdade de Filosofia Ciências e Letras de Ribeirão Preto, Brazil

## Abstract

The Florida harvester ant (*Pogonomyrmex badius*) excavates deep nests in the sandy soils of the Gulf and Atlantic coastal plains. Nest relocations of over 400 colonies in a north Florida coastal plains pine forest were tracked and mapped from 2010 to 2013. Individual colonies varied from one move in two years to four times a year, averaging about one per year. Almost all moves occurred between May and November peaking in July when more than 1% of the colonies moved per day. Move directions were random, and averaged 4 m, with few moves exceeding 10 m. Distance moved was not related to colony size. Over multiple moves, paths were random walks around the original nest location. Relocation is probably intrinsic to the life history of this species, and the causes of relocation remain obscure— the architecture of old and new nests was very similar, and neither the forest canopy nor the density or size of neighbors was correlated with relocation. Monitoring entire relocations (n = 20) showed that they were usually completed in 4 to 6 days. Moves were diurnal, peaking in the mornings and afternoons dipping during mid-day, and ceasing before sundown. Workers excavated the new nest continuously during the daytime throughout the move and beyond. A minority of workers carried seeds, charcoal and brood, with seeds being by far the most common burden. The proportion of burdened workers increased throughout the move. Measured from year to year, small colonies gained size and large ones lost it. Colonies moving more than once in two years lost more size than those moving less often, suggesting that moving may bear a fitness cost. Colony relocation is a dramatic and consistent feature of the life history of the Florida harvester ant, inviting inquiry into its proximal and ultimate causes.

## Introduction

On superficial inspection, it often appears that social insect colonies, especially ant colonies, live their lives rooted in place. Indeed, this observation has led several writers to make an analogy between ants and plants, observing that both are sedentary, compete in neighborhoods and grow (or shrink) by adding (or shedding) modules [1], [2]. Nests provide a defensible shelter with favorable physical conditions, whereas relocation expends energy and work, and exposes the colony to danger and stress. Nevertheless, reports of nest relocation accumulated gradually, so that by 1982, Smallwood [3] pointed out that relocation was common in ants. In his recent comprehensive review, McGlynn [4] concluded that natural selection has favored periodic nest relocation into the natural history of many, if not most social insects. He created 4 categories of nest relocation: legionary nomadism (such as seen in army ants), unstable nesting (e.g. Argentine ants), intrinsic nest relocation and adventitious nest relocation in response to disturbance. He also reviewed the many hypotheses that have been advanced to explain the evolution and maintenance of nest relocation. These include colony growth, spacing/competition, proximity to food, microclimate, nest deterioration or quality, sanitation/parasitism, predation and seasonality. Such causal hypotheses have rarely been tested through experiments. The reader is directed to the McGlynn review [4] for more depth. Clearly, nest relocation is part of a wide range of very different life histories, and probably arises through a range of selective factors, but it seems likely that many of the behaviors involved in moving the colony are common to most.

The tendency to move can be expressed in a number of ways— residence time in a location, probability of relocation per unit time, half-life of nests in a location and fraction of colonies moving in a specified time [3], [4]. The half-lives of ant species range from 13 to 2800 days [3], residence times from a few days or weeks to years, and percent moved per year from almost none to more than 100%.

Not surprisingly, the distance moved is loosely related to ant and colony size. Small cavity nesters and litter dwellers may move no more than a few cm (e.g. *Aphaenogaster rudis*, 40 cm; [3]), while leaf-cutter ant species with large colonies (*Atta sp., Acromyrmex sp.*) moved up to 320 m [5]–[8], some moving at night, others underground [9]. On the other hand, move distances may be rather modest even for some large ants, as for example the Florida harvester ant (the subject of this report) which moved an average of 2–6 m [10].

The time required to complete a move is also highly variable, and typically much shorter for cavity-nesters (minutes to hours) than for ground-nesters who have to excavate a new nest before or during relocation. Among the harvester ants, *P. barbatus* completed its move in 20–25 days [11], although Gordon [12] reported 1–2 days, an estimate that is probably in error. *P. badius* in South Carolina moved in 1 to 2 weeks, similar to the much larger nests of *Atta colombica* [5]. Reports that some moves of *Atta* sp. and *Acromyrmex* sp. were completed in 24 hr or even a single night [6] seem improbable, and need to be confirmed.

Nest relocation is obviously easier to study in cavity-nesters, so that much more is known of the causes, behaviors and organization of relocation in cavity-nesting ant species (e.g. [13]–[15] than in ground-nesting ones. However, because the present study is primarily concerned with the timing and parameters of relocations rather than with the behavioral mechanisms, this literature will not be further reviewed here.

Of greatest interest here are ground-nesting ants that exhibit McGlynn's [4] intrinsic nest relocation, that is, whose natural history includes periodic nest relocation, often for no readily apparent reason. Examples of soil-nesting ants that relocate include species of *Aphaenogaster*, *Pogonomyrmex*, *Messor*, *Polyrachis*, *Pheidole* and *Cataglyphis* (Table 1 in [4]). Relocation in ground-nesting ants clearly involves a great deal more labor, time, energy and risk than it does in cavity-nesting ants that move from one preformed cavity to another [16]–[20].

**Table 1:** Sampling dates and statistics of the multiply-sampled colonies.

Colony	Mean date of move, 2013	Total sample time, min.	Total observed period, min.	Percent of observed time sampled	Length of move, days	Final diameter of new nest disc
70	26-Jul	104	1027	10%	4.4	
85	28-Jul	272	2401	11%	7.3	49
152	19-Aug	264	2034	13%	8.1	46
217	11-Jul	222	2836	8%	8.3	43
246	19-Oct	136	1160	12%	11.2	
296	13-Jul	278	1981	14%	4.3	37
319	24-Aug	378	3434	11%	9.2	34
403	18-Oct	130	1047	12%	6.2	
428	16-Aug	126	1255	10%	7.1	36
441	15-Aug	164	1522	11%	6.1	40
496	28-Jul	224	2294	10%	8.1	31
539	19-Oct	202	1552	13%	14.2	

The Florida harvester ant is a characteristic, charismatic species of the Gulf and Atlantic coastal plains of the USA, ranging from Louisiana to North Carolina [21]. As the name implies, it harvests seeds and stores them in underground chambers for later consumption, much as most of the western North American species do. Relocation of the nest is an obvious phenomenon in part because it occurs frequently, in part because the nests are conspicuous and colonies do not move far. As a result, several reports describing relocation frequency, characteristics and possible causes can be found in the older literature [10], [11], [22]–[23].

In the course of mapping and monitoring a large population of *P. badius*, it became apparent that frequent remapping was producing an excellent record of who moves, how often, which direction and how far. Because relocations were so frequent, the entire process could be monitored from beginning to end.

## Materials and Methods

### Study site

The study population of Florida harvester ant, *Pogonomyrmex badius*, is located in a 23 ha site (latitude 30.3587, longitude −84.4177) about 16 km southwest of Tallahassee, Florida, USA, within the sandhills portion of the Apalachicola National Forest. The site, Ant Heaven, consists of excessively drained sandy soil occupying a slope to a wetland and stream, causing its water table to be depressed (>5 m at the maximum), thereby making it suitable for *P. badius* and *Solenopsis geminata*, as well as several drought-resistant species of plants such as *Opuntia* and *Nolina*. The area also supports a population of gopher tortoise (*Gopherus polyphemus*). The forest consisted of longleaf pines (*Pinus palustris*) planted ca. 1975, turkey oak (*Quercus laevis*), bluejack oak (*Quercus incana*), occasional sand pines (*Pinus clausa*) and sand live oak (*Quercus geminata*). Because the soil had been disturbed in the early 1970s, the natural ground cover of wiregrass (*Aristada stricta*) was absent, replaced by broomsedge (*Andropogon* spp.) and several other successional species of grasses, herbs and shrubs. The same disturbance may have helped establish this dense population of *P. badius*, whose nests are easily spotted because the ants decorate the excavated soil disc with a layer of charcoal bits (mostly the ends of burned pine needles) [24]. The black charcoal contrasts sharply with the light-colored sand or litter.

This project was carried out under US Forest Service, Apalachicola National Forest permit number APA56302, Expiration Date: 12/31/2017. *Pogonomyrmex badius* is not a protected species.

### Population mapping

**Figure 1:**
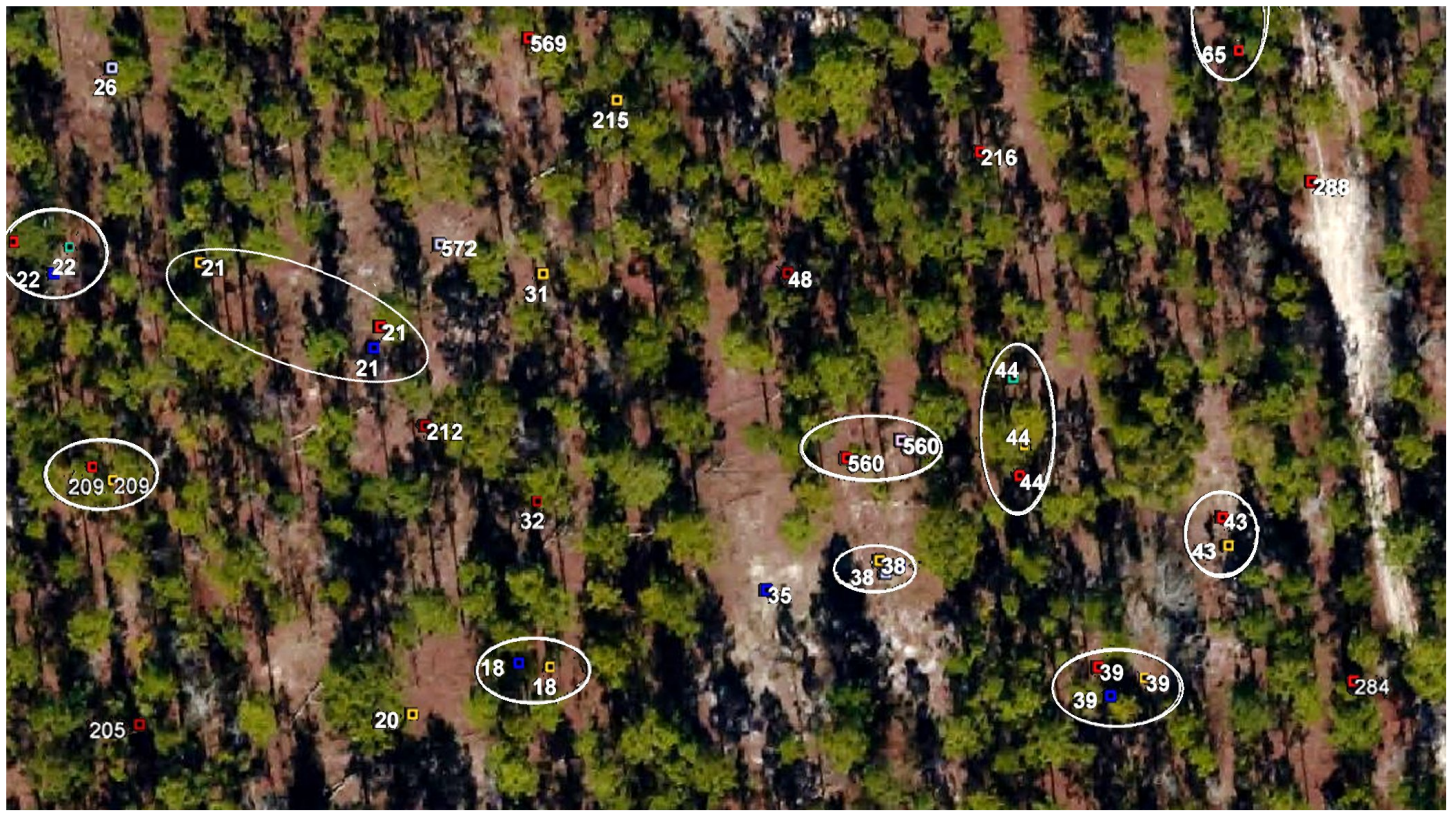
A representative section of Ant Heaven showing colony relocations during 2013 (enclosed in ellipses). Each 2013 survey is shown in a different color. Image from Leon County, Florida aerial survey in State Plane North, coordinate system GCS North American 1983 HARN. Images are public domain, funded by Leon County government.

This study required the detection of early stages of colony relocation. To facilitate this, each nest was marked with a vinyl flag and a numbered metal tag, and its location recorded on a Trimble GeoExplorer CE mapping GPS instrument. Location data were differentially corrected using the base station maintained by the Department of Environmental Protection in Tallahassee, resulting in a final precision of approximately 50 cm. In both 2012 and 2013 the population was resurveyed six times (i.e. every 4–6 weeks) between April and November in order to map completed or active relocations, inactive colonies and newly detected colonies (Fig. 1 shows a portion of the survey results for 2013). During surveys, areas between the easily visible flagged nests were preferentially searched to detect new or previously undetected nests. The population was first mapped in 2010, the mapped area enlarged in 2011. By the end of 2013, the tracked population numbered about 430 colonies.

During the summer of 2013, the population was checked daily until 3 colonies that had just begun to excavate a new nest were detected. These colonies were then observed until their move was complete, whereupon the general population was again checked daily. This routine was repeated between June and late October. A total of 20 relocations was tracked.

### Observation of moves in progress

Relocations in an early stage were recognizable by the appearance of a new, small nest disc connected to the mapped colony by an active, 2-way trail. In a few cases, two or even three new nests were started, but in all cases, all but one were soon abandoned. The move was then quantified as follows: an arbitrary point about halfway between the old and the new nest disc was designated, and 2-minute counts were taken of the number of dark (older) workers walking to the new and old nest, the number carrying brood, seeds or charcoal, and the number of callow (young) workers walking to the old and new nests. Workers and carried items were counted in separate 2-minute counts, the latter following immediately upon the first. The primary data were thus the movement and direction of ants and items per minute. Each count was also accompanied by a measurement of the infrared temperature (Westward Brand, range −50 to 500°C., accuracy +/−1°C.) of several points of the trail, and notes of the weather and sun/cloudiness were made. During the first 8 relocations, single 2-minute counts of each type of item were made 20–45 minutes apart, but during the later 12 relocations, counts were made in groups of 3 spaced about 5 minutes apart, with groups spaced 30–45 minutes apart.

Most analyses were based on the means of the 3-count groups. On the assumption that these mean rates applied until the next set of counts, an estimate of the total movement of ants or items from one set to the next was made by multiplying the mean rate by the minutes elapsing between sets. When large intervals between sets occurred (e.g. night time, or a mid-day lull), an elapsed time of 20 minutes was used. The sum of these products was used to estimate the “observed total” per day or per move. By computing the fraction of the observed total moving per time unit (day or fraction of day) all moves were converted to the same scale. The “observed total” was an underestimate of the true totals because moves were not observed during their entirety. The period during which an observer was present and made counts is referred to as the “observed period.” Sampling statistics are given in Table 1.

### Excavation and mapping of nests

Excavation and chamber mapping methods were similar to those of Tschinkel [25]. A pit large enough to work in was dug next to the focal nest, and the chambers exposed one by one by lifting off the soil in horizontal layers to expose the outlines of each chamber. A battery-powered shop vacuum was used to collect chamber contents, and to clear remaining sand from chambers before a sheet of clear acetate was laid over the chamber and the chamber outline traced. The tracing was located in an x,y,z grid by noting its coordinates in cm in reference to a 0, 0, 0 point on the ground surface. All collected nest contents were later counted in the laboratory. Together, these data allowed the 3-dimensional reconstruction of the nest chamber arrangement and nest contents as in Tschinkel [25]). After excavating and mapping, colonies were allowed to re-establish a nest at their original location. Colony survival was excellent, and colonies appeared regularly in subsequent surveys.

### Data analysis

Continuous variables were analyzed by regression or ANOVA as appropriate, and transformed to stabilize the variance where necessary. Count data were analyzed by Chi-square or other non-parametric tests. Because the number of colonies tracked increased during the course of this study, care was taken to use the appropriate n for computing all statistics.

The basic data on which this analysis is based can be found in Table S1.

## Results

**Figure 2:**
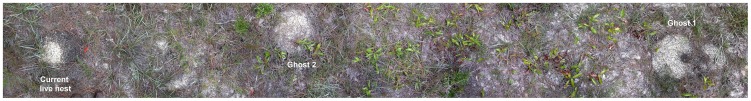
A currently-occupied nest (left) and two previously vacated nests (ghosts) of a colony of *Pogonomyrmex badius*, the Florida harvester ant. Up to 4 or 5 ghosts may be visible in the vicinity of some colonies. Ghosts often remain visible for more than a year until they are gradually leveled and dispersed by wind and rain, and covered with litter.

The Florida harvester ant, *Pogonomyrmex badius*, is an excellent subject for the study of nest relocation by a soil-nesting ant for several reasons: (1) the distance moved is much less than the typical distance separating the neighbors, greatly reducing uncertainty that a newly appeared nest is occupied by the colony vacating the old nest (Fig. 2); (2) the live nest discs are decorated with bits of charcoal, making them conspicuous, and the charcoal is transported to the new nest during relocation; (3) the discs of vacated nests (ghosts) are visible for months or even years (Fig. 2); (4) colonies move about once a year on average; (5) flagging and numbering live nests and revisiting them several times a year produces an unambiguous record of relocations and allows colonies to be tracked over many years.

**Figure 3:**
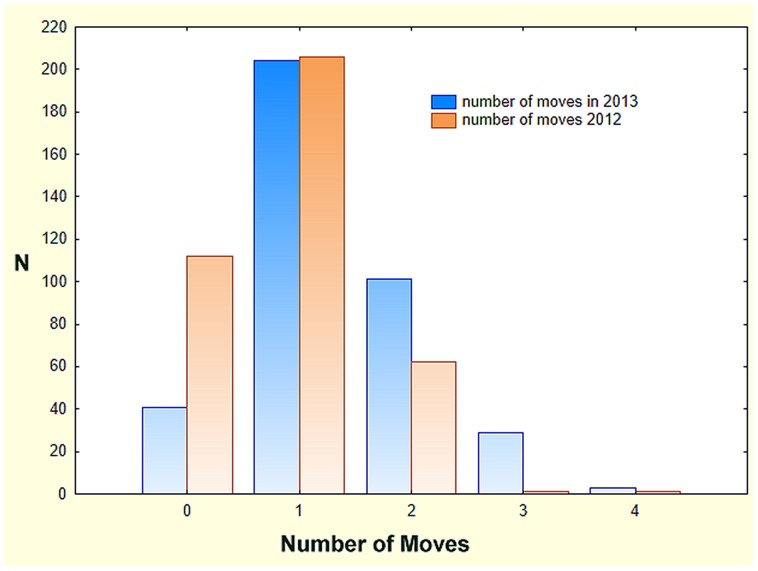
The frequency distribution of the number of moves by colonies in 2012 and 2013. The increase in the number of moves in 2013 compared to 2012 resulted from a decrease in the number of colonies that did not move, and an increase in those that moved multiple times.

During 2012–13, 473 colonies were observed to relocate 841 times (Fig. 3). During 2012 colonies moved an average of 0.72 times, but in 2013, the average was 1.15 times, significantly higher (t-test: t_903_ = 8.27, p<0.00001), The frequency distributions were significantly skewed in both years as many colonies moved multiple times (skew = 0.54 and 0.64, respectively, s.e. = 0.11). In both years, about 200 colonies moved only once. The higher mean in 2013 was the result of a decrease in the number of colonies that did not move, and an increase in those moving more than once, with some colonies moving up to 4 times.

**Figure 4:**
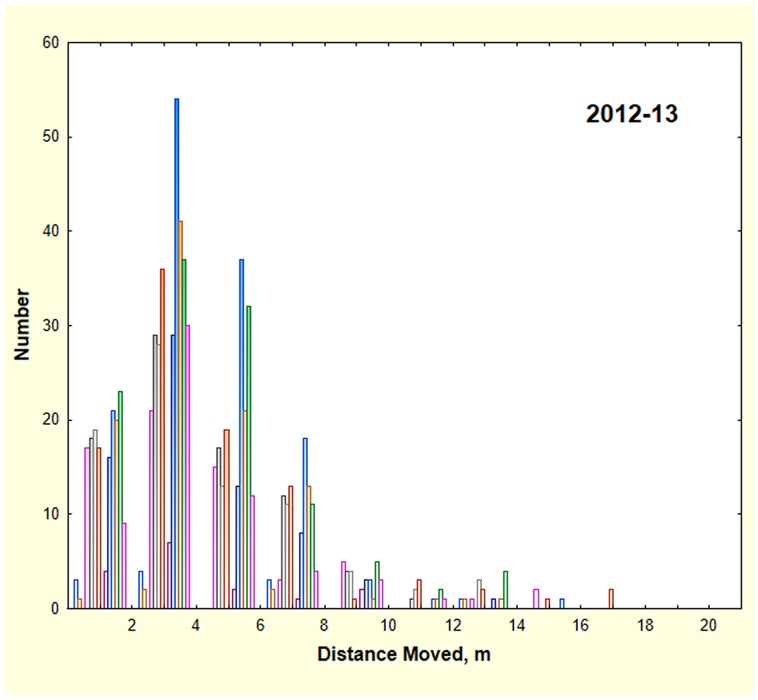
The frequency distribution of the distance moved by colonies in 2013. Each 2013 survey is shown in a different color. The distribution is right-skewed, but the differences between surveys were not significant.

The distance colonies moved averaged 4.1 m (N = 811). The distribution of distances was skewed to the right, with a few colonies making moves of 8 m or more (Fig. 4). During 2012–13, the mean distance moved during each of the 10 surveys with more than 10 moves (surveys are shown in different colors in Fig. 4) ranged from 3.7 to 4.4 m, but these differences were not significant. A representative section of Ant Heaven can be seen in Fig. 1, showing that colonies had 1 to 3 locations during 2013. Colony size, as estimated from disc diameter, was not related to distance moved (ANOVA, n.s.).

**Figure 5:**
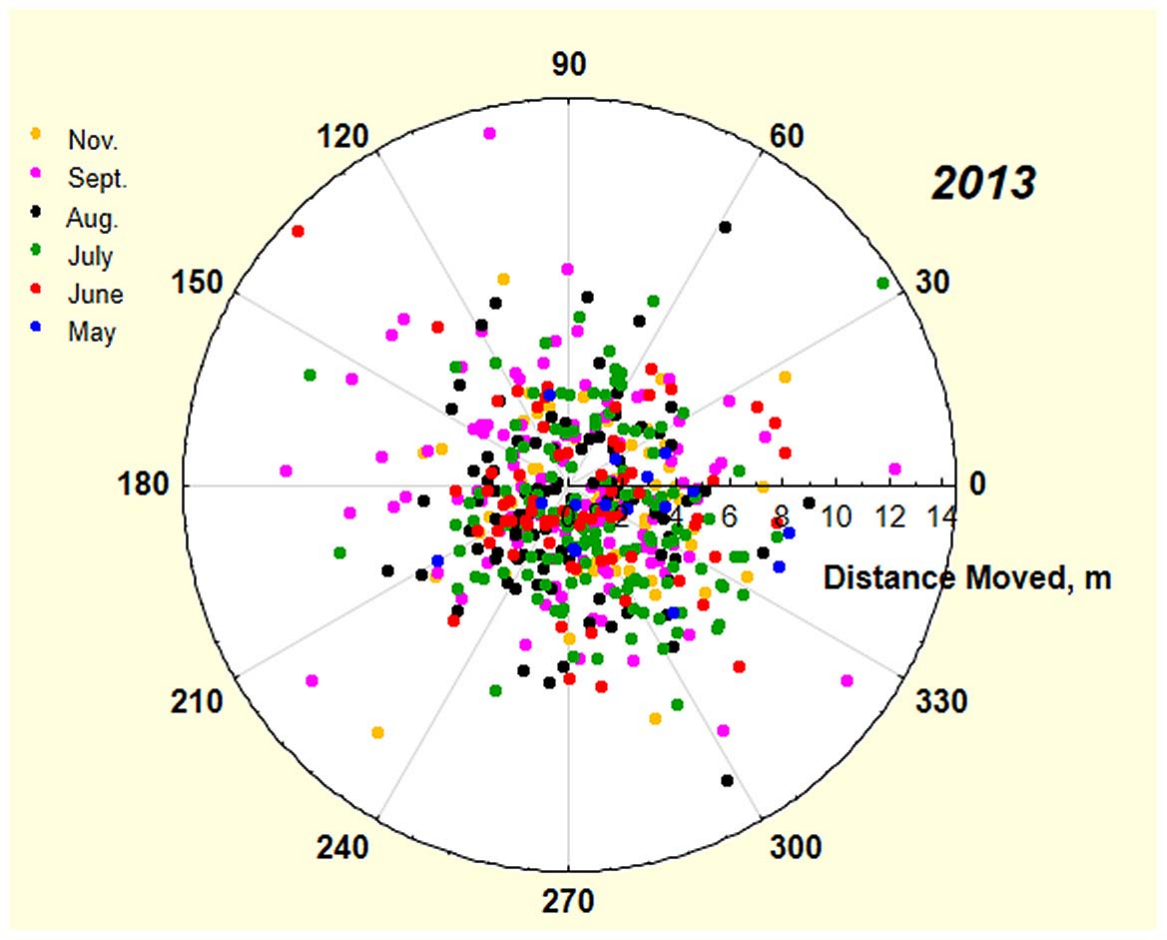
The direction and distance of moves by colonies in six surveys in 2013. The azimuth of moves was random across the population and for each survey. The date of each survey is shown in different colored symbols.

At the population level, the compass heading of the relocations was random during every survey (Fig. 5). However, it is possible for moves to be in random directions during each survey while individual colonies have a preferred direction. Nevertheless, individual colonies showed no consistent preferred direction of movement, nor did they consistently move away from their nearest neighbor.

**Figure 6:**
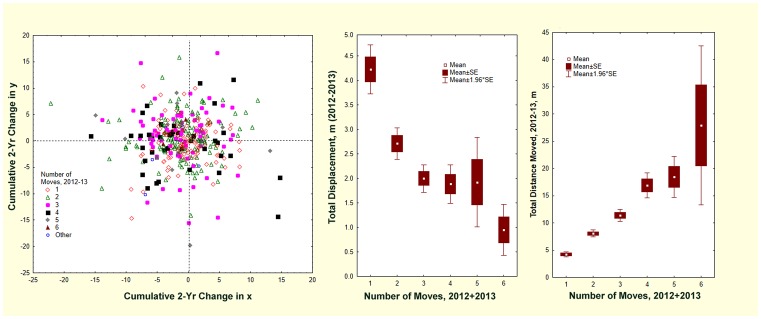
The displacement of nest position (x,y coordinates) in relation to the number of moves by colonies in 2012 and 2013. The more often colonies moved, the greater the distance they traveled and the closer to their original location they ended up. In essence, over multiple moves, colonies perform a random walk around their initial position.

A relocation results in a positive or negative change in the x and y coordinates, so that the new nest is displaced in space from the old. By cumulating the changes in x and y over all moves during 2012–13, the final displacement of the November 2013 nest relative to the initial April 2012 nest was revealed (Fig. 6A, B). The mean total distance moved was simply a multiple of the mean distance moved (i.e. 4.1 m, Fig. 6C), but the displacement from the original position was negatively related to the number of times a colony moved (Fig. 6B). Combining these results with the random direction of the moves (Fig. 5) suggested that multiple moves result in a “random walk” around the original nest location. The more often a colony moves, the closer to its starting location it is likely to zig and zag. Thus, colonies that moved 6 times (in 2 years), covering a total mean distance of 28–30 m, ended up within less than a meter of where they started.

### Seasonality of moves

In 2012 and 2013, surveys were conducted more or less monthly except during winter. The number of active colonies ranged from 310 to 405, and the number of relocations per survey ranged from 2 to 138. Relocation rates were computed for each survey as the fraction of active colonies that moved, divided by the days elapsed since the previous survey (moved/total active/day). This can also be understood as the daily probability of moving. Although Smallwood [3] also suggested estimating “residence time” and “half-life” at a location, these are meaningful only if they are fairly constant over time, whereas the relocation rate in *P. badius* was strongly seasonal.

**Figure 7:**
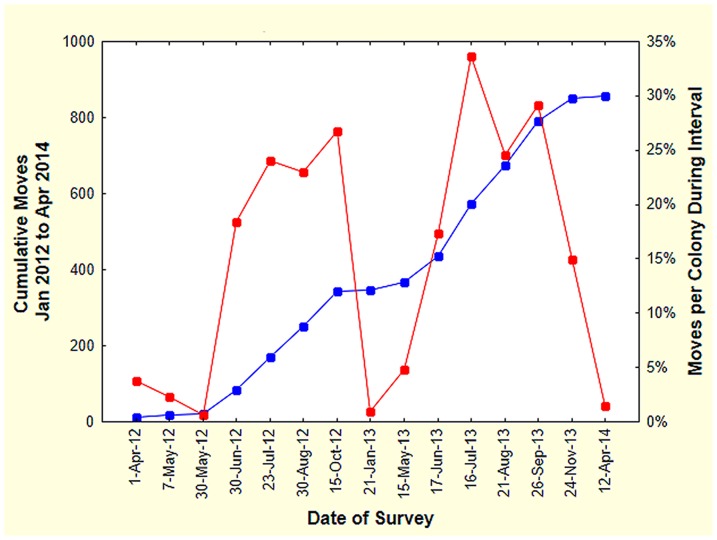
Seasonality of relocation. During July and August of each year, over 1% of the active colonies moved per day. Moves were rare between December and April, the “winter” in Florida. Cumulative moves over the two years is shown on the left scale, and percent moving between surveys on the right scale.

Colonies moved primarily during the warm season between late May and early November (Fig. 7). In both years, the move rate increased from very low rates before June to peak in July when more than 1% of the colonies moved every day (i.e. the probability that a given colony would move on a given day was 1 in 100). The suppression of the rate during August in both years may be real, for the frequent rains in August may inhibit relocations. Declining temperatures in November and December reduced the relocation rate to winter levels. Nevertheless, a handful of moves usually occurred between early December and April.

### Possible causes of relocations

**Figure 8:**
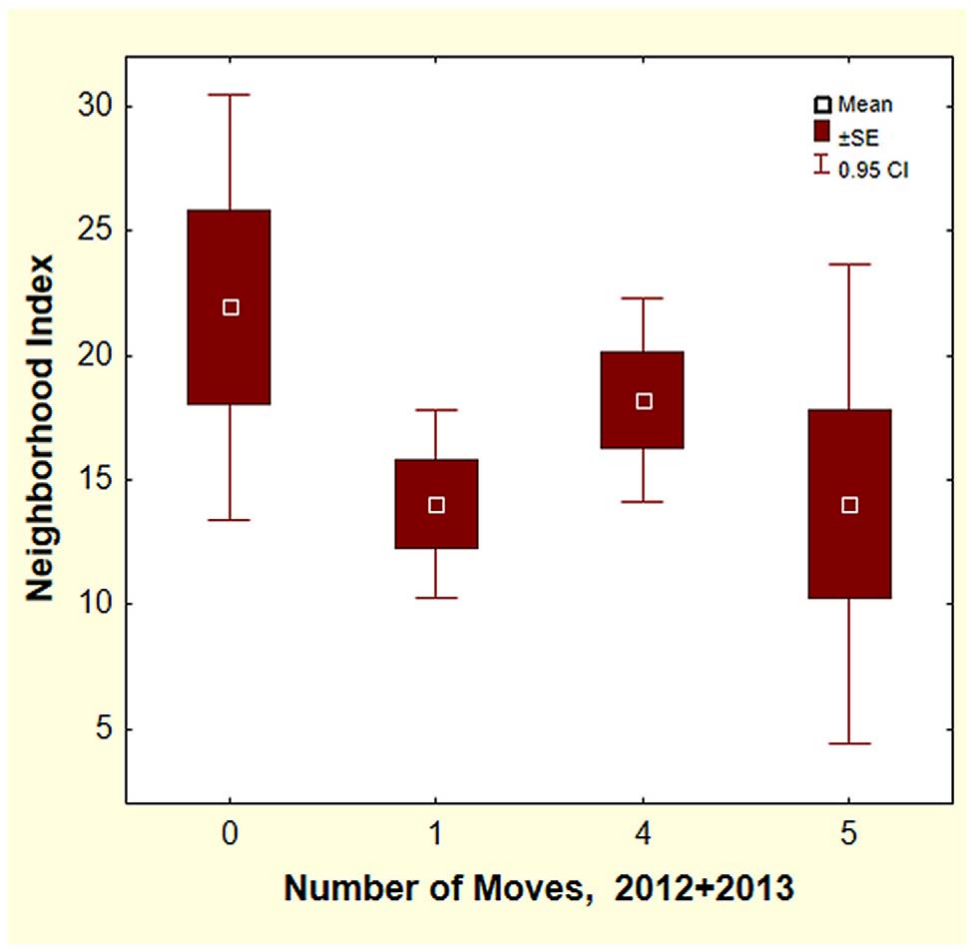
The number of moves was not related to the size and number of neighbors (computed as a neighborhood index (see text). Only colonies moving few times (0 or 1) or many times (4 or 5) were tested. Exclusion of newly-appeared colonies in 2013 reduced the differences even more.

Why do colonies relocate their nests? Perhaps they are responding to interactions with neighboring colonies and relocation reduces this interaction. If this is so, then colonies with larger and/or closer neighbors should move more often. A “neighborhood index” was computed as follows: size of neighbors was estimated from the area of the nest disc (which is proportional to the total nest size). The effect of neighbors can be expected to decrease with the square of their distance from the focal colony. The neighborhood index was the sum of the disc areas of the first three neighbors each divided by the square of its distance from the focal colony. The distance to the first neighbor averaged 14.6 m, to the second 18.4 m and the third 22 m. The neighborhood index ranged from 5 to 52 but was not related to the number of moves in 2012–13 (Fig. 8. One-way ANOVA; F_3,46_ = 1.99; n.s, using 0, 1, 4, 5 moves, i.e. the extremes). A pairwise inspection of moves with nearest neighbors found no evidence that colonies move away from possible competitors.

The physical characteristics of the nest location might also influence the restlessness of a colony— we can expect that there are optimal combinations of sun exposure, soil moisture, vegetation and so on. Using Google Earth images, the study site at Ant Heaven was divided into blocks based largely on past management practices and timing that resulted in visible differences in canopy density, ground cover and litter density. The percent canopy cover of each block was determined from the images, and ranged from 42% to 72%. The number of colonies making from 0 to 6 moves within each block was counted and compared to the expected number, which was calculated from the fraction of the colonies in the entire site that moved 0 to 6 times.

Colonies did not move more or less frequently in any of the blocks (Chi-Square = 67.48; df = 69; p = 0.53, n.s.). Whatever environmental differences existed among the blocks was not associated with the tendency to move frequently.

It is also possible that the colony outgrows its nest and constructs a new, larger one. However, this is not supported by excavation and mapping of the old and new colonies. The total area of the new nest averaged about 18% smaller (s.e. = 11; n = 6) and the maximum depth about 5% less (s.d. = 26%, not significantly different from zero). Both of these differences were probably the result of the nest excavation not yet being complete at the time of mapping (usually less than 2 weeks post-move).

**Figure 9:**
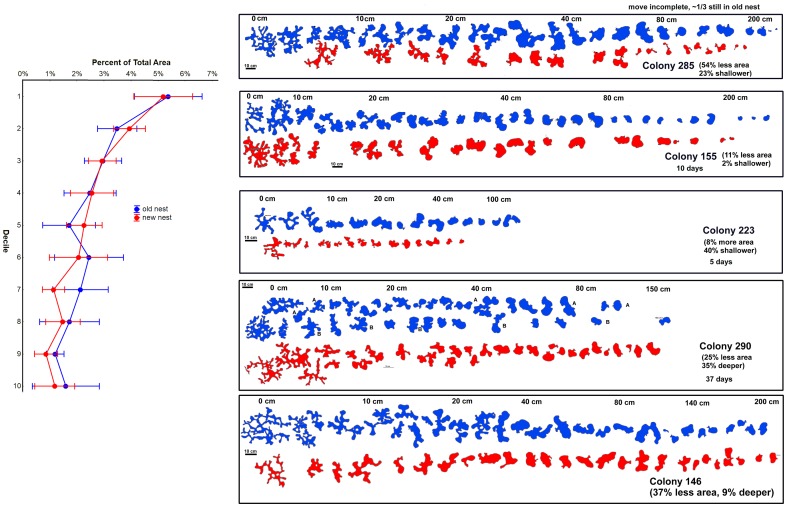
The new and old nests of relocated colonies differed little in size, depth, chamber shape and chamber distribution. Five examples are shown. The approximate depth of the chambers is shown by the scale in each panel. The 10 cm scale applies to chamber size. The notes associated with each colony number records the relative size and depth of the old and new nests, and the duration of the move. The graph at the left shows that in old and new nests, the proportion of total area is distributed similarly in relation to the proportion of total depths (deciles). Other measure of nest “shape” were also similar.

In addition to the small size differences between the old and new nests, several size-free shape measures [24] remained the same (Fig. 9). The proportion of the total chamber area in each decile was essentially identical between the old and new nests (Fig. 9), as was the vertical distribution of chamber circularity (not shown). These observations are in line with Tschinkel's [26] report that several size-free shape estimates of *P. badius* nests remained constant as nests grew, that is, the nest “shape” did not change.

### The cost of moving

**Figure 10:**
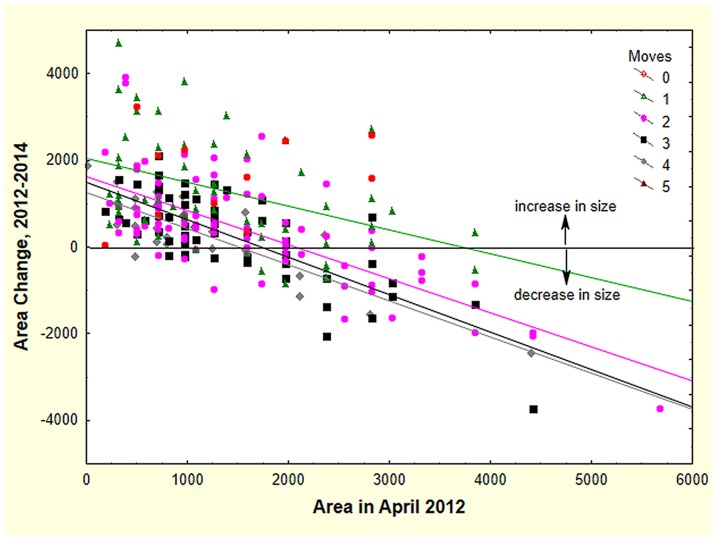
Initially large colonies tended to lose size, and initially small colonies tended to gain size (as measured by disc area). Colonies that moved more than once lost size more rapidly. The slopes for colonies moving 2 to 4 times did not differ significantly, and lost about 0.8 cm^2^ for every cm^2^ of initial size. Colonies moving only once lost significantly less, about 0.5 cm^2^ per initial cm^2^.

If moving exacts a fitness cost from the colony, this cost might be detectable as changes in colony size (a proxy for fitness). Colony size was estimated for both 2012 and 2013 by comparing the nest disc size (which is correlated to colony size: log worker number = −0.44+1.20×log disc area) of moved colonies during the same month one year later (e.g. April to April) and two years later (April 2012 to April 2014). Fig. 10 shows that colonies that were small in 2012 mostly increased in size over two years, and those that were initially large lost size (multiple regression with dummy variables and interaction terms: F_9,244_ = 22.81; p<0.00001; R^2^ = 44%). Most of this size change was probably a consequence of growth in small colonies and sexual production in large ones (as documented in the fire ant, *Solenopsis invicta* [27]). If moving exacted a cost, then colonies that moved more often should lose size more rapidly. Colonies that did not move did not decrease significantly in size (but this sample size was small). Colonies that moved once lost about 0.5 cm^2^ for each initial cm^2^, significantly greater than zero (t _245_ = −4.09; p<0.00001), and this rate increased significantly to about 0.8 cm^2^ per initial cm^2^ for colonies that moved 2 to 4 times (t _245_ = −2.25; p<0.05). Colonies moving 5 times showed no significant decrease, but the sample size was small. Overall, the increased rate of size loss with increased relocation frequency is consistent with a cost of moving. Of course, unknown correlates of moving could also be responsible for this phenomenon.

#### The process of moving

**Figure 11:**
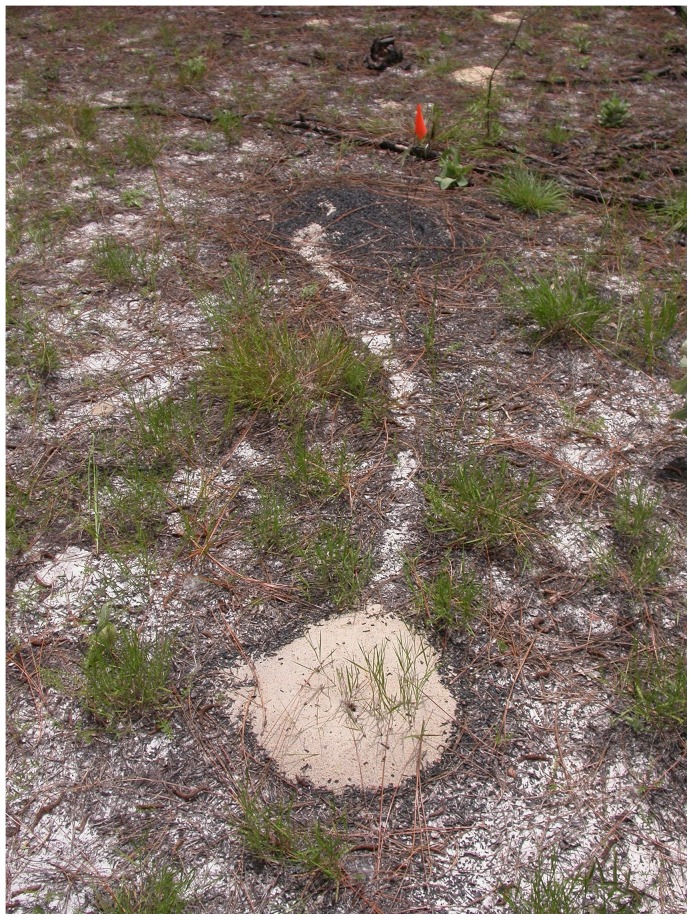
Colonies sometimes cleared visible trails during moves. The charcoal covered nest disc is the old nest, and the clear, sandy one is the new.

**Figure 12:**
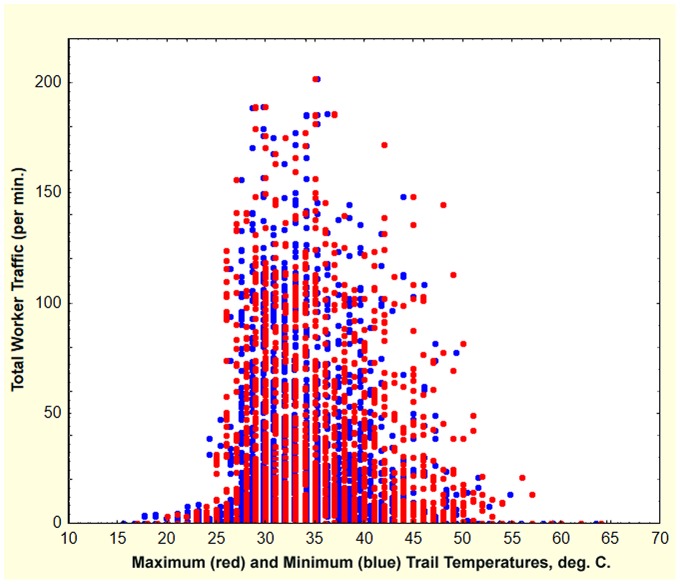
Maximum (red symbols) and minimum (blue symbols) trail temperatures in relation to total bidirectional worker traffic (workers per minute). Most traffic occurred when trail surfaces were between 25 and 45°C. Temperatures were measured with an infrared thermometer.

Relocations in their early stages were identifiable through the very small nest disc around the new nest, and a trail between the old and new nests. This early trail was usually much less active than later in a move, at which time the ants occasionally formed conspicuous cleared trails between the new and old nests (Fig. 11), but most trails were not conspicuously cleared. During every day of the move, workers at the new nest brought pellets of sand to the surface and deposited them on the growing nest disc. All trail activity began no earlier than about 8:30 or 9 a.m. and ceased no later than about 6 or 7 p.m. Trail activity often diminished or ceased during the hottest part of the day (noon to 3 p.m.). Substantial rain inhibited trail activity, but light rain often did not. In all cases, trailing and excavation ceased before sunset at which time the ants closed the nest entrances of both nests, to resume surface activity the next morning Trails were most likely when the minimum surface temperature on the trail was above about 25°C and the maximum was less than about 45°C (Fig. 12).

**Figure 13:**
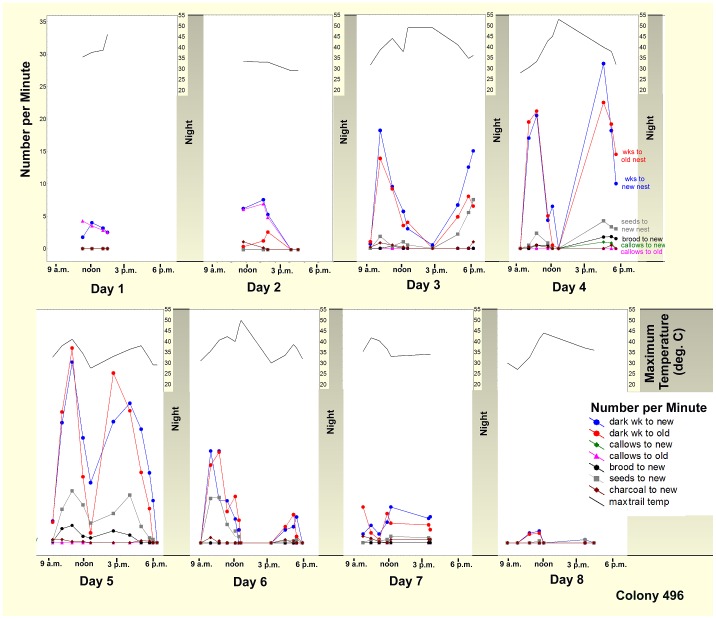
The daily and hourly progress of a representative move. Rates per minute are the means of 3 closely spaced, 2-minute counts, with groups of counts 30–45 minutes apart. Traffic usually slowed during the mid-day temperature maximum (upper grey line and scale). Peak traffic increased day by day until days 4 and 5, and then declined to lower levels for the remainder of the move, which in this case took 8 days. Different colored lines indicate the rates of workers to and from the new nest, and brood, seeds, callows and charcoal to the new nest. These data are summarized in several ways for all the multiply-sampled colonies in Figs. 14–17.

The daily patterns of bidirectional trail activity and transport were estimated as items per minute passing a fixed point on the trail. Fig. 13 shows a representative move during July 2013 and reveals several features common to most moves. The rate of workers moving to and from the new nest was usually very similar, with both peaking in the late morning, decreasing or ceasing during the mid-day temperature maxima, rising to a second peak in the late afternoon, before ceasing in the evening. The peak rates increased until the fifth day and then dwindled until the move was complete on Day 8. The rates of brood, seed and charcoal movement to the new nest were much lower than that of workers, indicating that most workers made the trip to the new nest unburdened. Seeds, brood and charcoal were almost never carried from the new to the old nest, and are omitted from the analysis.

**Figure 14:**
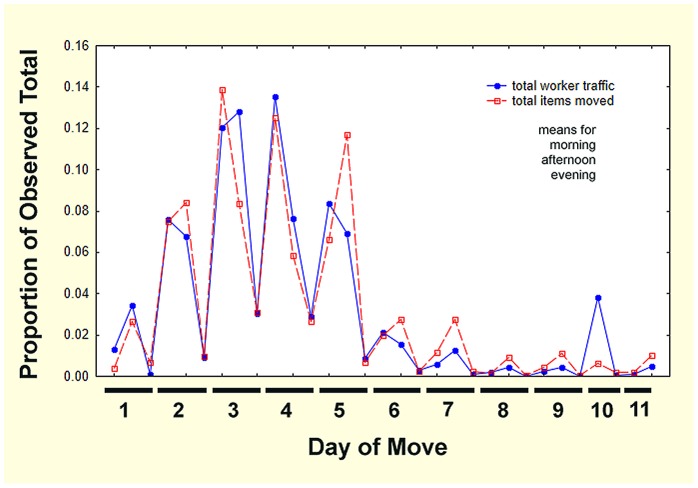
The moves of 12 colonies normalized as proportions of the observed totals moved during three time periods over 10 days. This presentation removes the effect of differences in colony size and move rates, revealing the shared pattern of move progress.

Combining the data for all colonies and normalizing as proportion of observed totals revealed a strong daily and move-length pattern. Fig. 14 shows the fraction of the observed total occurring during three broad time periods of each day (morning, afternoon, evening) for the duration of the move. For this figure, worker traffic in both directions was summed as total worker traffic (including callows), and all carried items (brood, seeds, charcoal) as total items moved. All traffic increased to peak at Day 4 or 5 and then gradually declined. On average, traffic did not drop to zero during the peak days of the move because traffic started earlier and ended later during this period.

Items were carried to the new nest throughout the move. Most moves were complete by Day 6 or 8, but one continued until Day 11 and another Day 14 (not shown in Fig. 14). However, in no move was traffic heavy after about 6 or 7 days. Interestingly, while the majority of workers made the trip to the new nest unburdened, the proportion of the items moved remained parallel to the worker traffic, suggesting that movement of items is simply proportional to worker movement (but see below).

**Figure 15:**
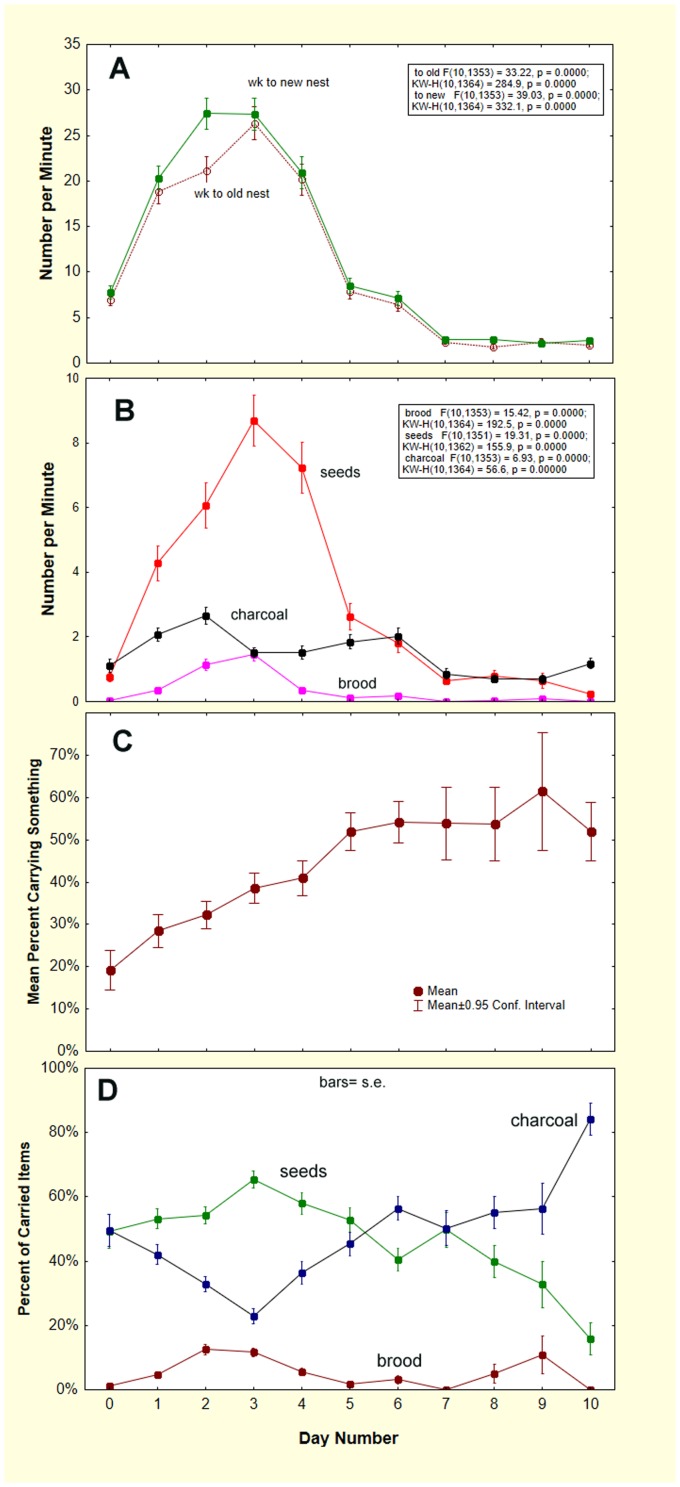
The daily progress of moves. A. workers per minute to and from the new nest; B. items carried to the new nests per minute; C. Percent of workers carrying something; D. Items as percent of total carried.

The average rates of movement (per minute) day by day can be seen in Fig. 15. Worker movement rate to the new nest predominates slightly over that to the old, as should be the case when a colony moves from one nest to the other (Fig. 15A). The average movement of seeds lags worker movement slightly, but is more or less proportional to worker movement, as is the movement of brood (Fig. 15B). Summing brood, seeds and charcoal as “carried something”, Fig. 15C shows that the proportion of workers going to the new nest “carrying something” increased gradually throughout the move, while at the same time, worker movement peaked and declined to low levels. As a proportion of the items carried, charcoal increased at the expense of seeds later in the move (Fig. 15D). Brood peaked somewhat earlier than seeds, and showed a smaller, more variable peak late in the move.

**Figure 16:**
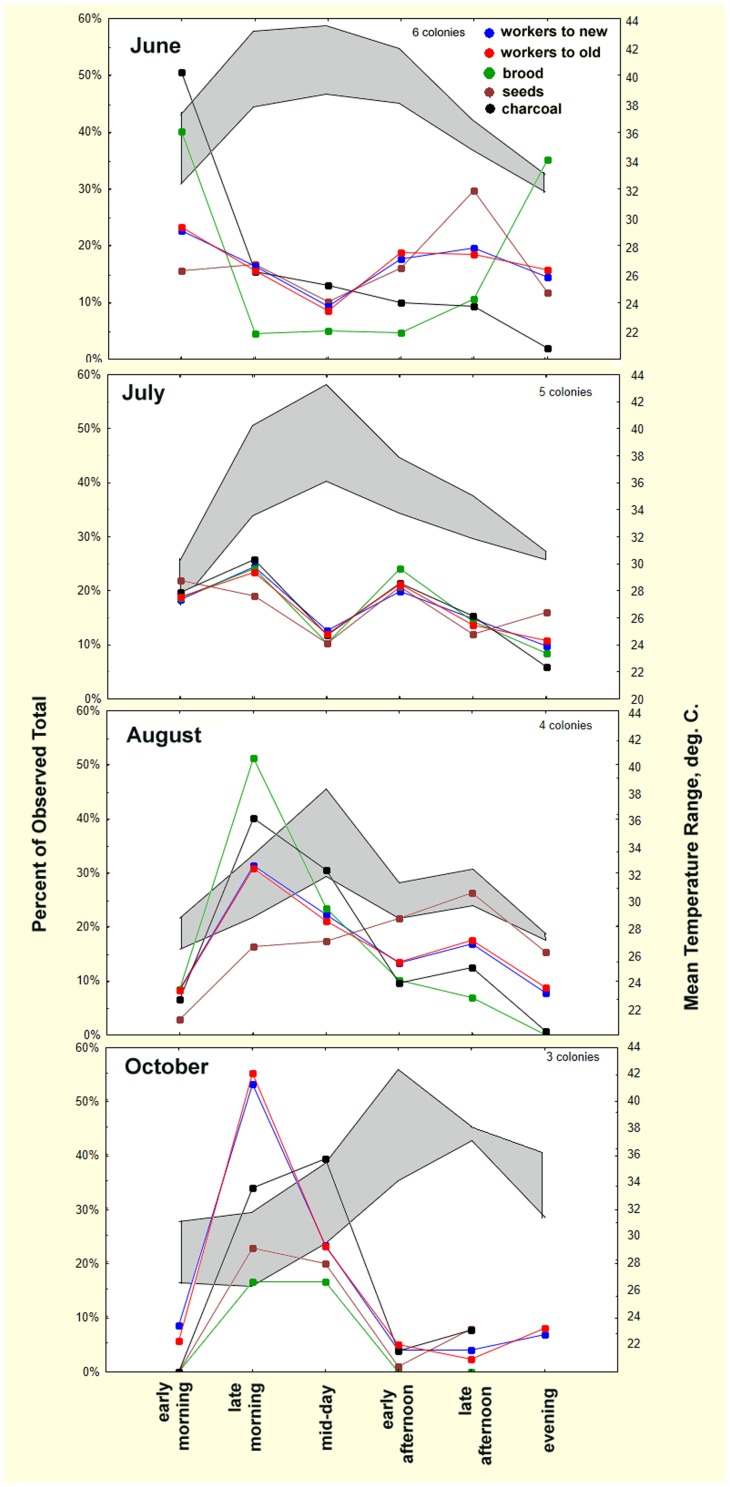
Average normalized activity by time of day (in 2-hr blocks) in four sampled months of 2013. The grey zone shows the mean range of trail temperature by time of day. The ordinate shows activity as proportion of the observed total. Peak activity occurs early and late in the day in June, but shifts to mid-day by August and October.

The large differences among colonies in the rates of worker, brood, seed, callows and charcoal movement suggest that normalizing the data as fraction of the total observed items or ants moved during 2-hr time blocks between 8:30 a.m. and 8:30 p.m. would put colonies of different sizes and rates on the same scale. Fig. 16 presents such data for the daily movement activity over the season atop the mean trail temperature range. June temperatures were high much of the day, probably because the weather was very sunny. Between June and October, peak temperatures occurred later in the day. Across the season, the fraction of items and ants moved during each 2-hr period changed dramatically. Worker traffic to and from the new nest showed a distinct mid-day decrease in June and July. August's greater cloudiness and more frequent rain reduced the maximum temperatures, reducing early morning activity, allowing a greater proportion of movement mid-day, and moving the lull to early afternoon. By October, mornings remained cool longer and maximum temperatures occurred later in the day, reducing early morning activity and creating a large peak in traffic during late morning and mid-day. Across months, evenness of traffic throughout the day decreased after July, with a sharper, larger peak occurring during late morning, and decreased activity after mid-day.

The movement of items carried to the new nest by the workers did not parallel worker traffic in 3 of the 4 months. In June, brood and charcoal were moved preferentially early in the day, and seeds late. July was the only month in which all items were moved parallel to worker traffic. By August, brood and charcoal were once again moved preferentially in the morning (though not quite as early as June), and seeds late in the day. With October's lower temperatures, movement of all items peaked in the late morning and mid-day, dropping to low levels later in the day.

**Figure 17:**
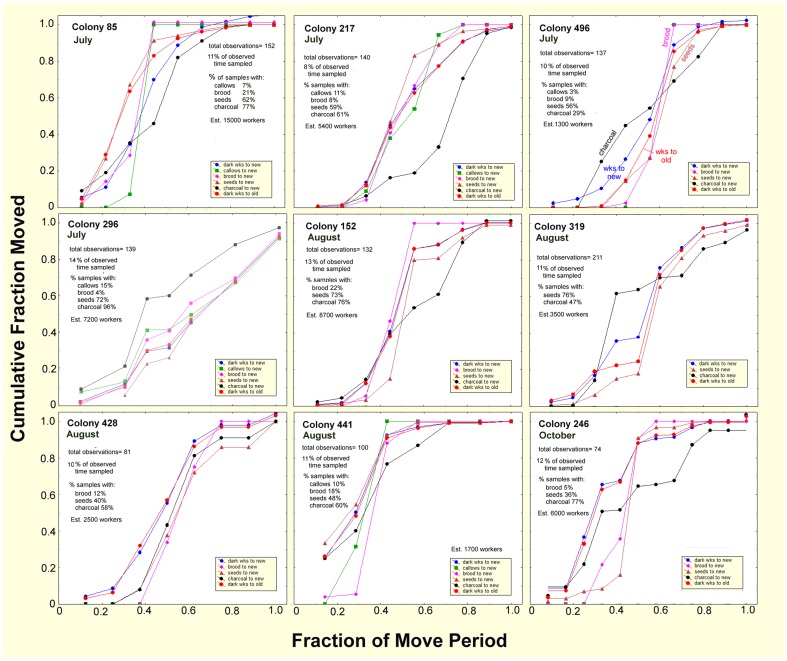
The progress of 9 moves in relation to the proportion of the move completed. The ordinate shows the proportion of the observed total workers or moved items. These double-normalized plots reveal the size-free and duration-free patterns of the timing of moves. The legends for the different colored lines are shown in the insets.

Fig. 17 depicts cumulative moves normalized on two scales— the proportion of the observed total items or ants moved, and tenths of the total move time completed. Differences among colonies with respect to the rate and timing of moves can be seen through the cumulation of items as the move progresses. Items reach 100% at different proportions of the move (items seen in fewer than 4% of the samples and/or with estimates lower than 100 are not shown in Fig. 15). Items moved continuously by workers cumulate at the same rate as the workers and those moved early or late do not. The slope of the cumulative curves reveals whether the item was moved gradually and continually, or in brief bursts.

Fig. 17 shows several distinct patterns among the colonies. In general, the slow start, buildup to a maximum and tapering off seen in Fig. 14 can be recognized in the sigmoidal nature of most of the curves in Fig. 17. In most colonies, movement of seeds was parallel or nearly so with worker traffic, indicating that seeds are transported at similar rates throughout most of the move. Colonies 246, 428 and 496 show a distinct lag before the transport of seeds picked up. The transport of charcoal can either precede (colonies 496, 296, 319) or lag (colonies 85, 217, 152, 246) the general movement of workers. Seeds were usually transported nearly continuously, cumulating more or less in parallel with worker trips (although colonies 246 and 428 seemed to show a lag). Several colonies did not have enough brood or callows to assess their movement. In colonies with callows (colonies 85, 217, 496, 296, 441), these made the move to the new nest over a brief span of time, mostly 10–40% of the length of the move. Only in colony 296 did callows move more or less in parallel with workers. Transport of brood showed similar patterns, with most transport occurring in 20–40% of the length of the move. Exceptions included colonies 217 and 296. Although it might be expected that the transport of seeds, brood and charcoal might be negatively dependent upon one another, this effect was probably weak, as only 12 to 46% of the workers on the way to the new nest were burdened.

The cumulative patterns noted above were also apparent in the fraction of 2-minute counts in which items were seen (i.e. non-zero counts). The mean number of samples for the 12 multi-sampled colonies was 122 (s.d. = 42; range 70 to 211). Seeds were seen in 57% of these (range 30–92%) and charcoal in 62% (range 29–77%), but brood were seen in only 12% (range 2–22%) and callows in 6% (range 1–15%; 4 colonies lacked callows). These differences resulted in very large differences of the estimates of the observed total of items transported (Table 2).

**Table 2:** Summary statistics for the multiple-sampled relocations.

	Mean workers per min		Maximum workers per min.		Total workers during observed period		Net workers to new nest	total estimated callows	total estimated brood	total estimated seeds	total estimated charcoal
Colony	to new nest	to old nest	to new nest	to old nest	to new nest	to old nest		to new nest during observed period	to new nest during observed period	to new nest during observed period	to new nest during observed period
70	3.1	3.1	13	13	3464	3554	−90	0	0	91	23
85	12.6	11.0	65	64	35256	31390	3866	160	1146	7568	5989
152	24.4	23.0	100	98	45285	42961	2324	255	2079	8714	5613
217	11.0	8.9	45	47	32607	26387	6220	501	1747	6131	5223
246	9.0	8.4	37	51	11085	10084	1001	0	142	2354	2491
296	30.6	30.9	100	68	65746	66765	−1019	293	1018	32329	4786
319	17.8	12.9	97	92	65480	51087	14393	30	91	20496	4190
403	12.2	10.7	65	60	13885	12385	1500	0	145	2076	1778
428	5.9	5.0	30	26	7663	6905	758	0	510	347	1662
441	10.8	10.8	47	58	15967	15734	233	72	1250	784	2163
496	6.6	5.4	32	39	17548	15146	2402	58	725	3778	554
539	4.6	4.1	27	24	8411	7341	1069	16	312	680	941

#### Summary statistics

Summary statistics by colony help to emphasize some of the features of relocations (Tables 2 and 3). Worker traffic to and from the new nest was usually very similar, sometimes with net movement to the new nest, sometimes not, and sometimes the reverse. Worker counts in each direction varied from 0 to about 200 per minute. The mean rates were 12.4 per min. to the new nest, and 11.2 per min. to the old, assuring that on average, the worker population in the new nest gradually increased at the expense of the old. Each worker no doubt made multiple trips. During the observed period, the estimated number of trips to the new nest ranged from about 3500 to 65,500, and averaged 32,100 (n = 12). The estimated reverse trips ranged from 3500 to 67,000 and averaged 28,400. Much of this variation can probably be attributed to differences in colony size. *P. badius* colonies rarely have more than about 8,000 workers, making it clear that on average, workers make multiple trips back and forth during the moves. Given an average colony size of about 4,000, each worker made about 7 to 8 trips, but undoubtedly the number of trips is not evenly distributed over the worker population. It seems likely that foragers make more trips than other workers, but this needs to be tested.

**Table 3:** Summary statistics for transported items by colony.

Colony	mean % with seeds	mean % t with brood	mean % with charcoal	mean % carrying something	% of carrying with seeds	% of carrying with brood	% of carrying with charcoal	mean trips per brood	mean trips per seed	mean trips per charcoal
70	2%	0%	5%	7%	53%	0%	47%		38.2	
85	17%	2%	1%	20%	87%	6%	7%	30.8	4.7	5.9
152	22%	3%	0%	26%	81%	18%	1%	21.8	5.2	8.1
217	18%	3%	1%	22%	80%	15%	5%	18.7	5.3	6.2
246	15%	1%	2%	18%	82%	6%	12%	78.3	4.7	4.4
296	44%	1%	1%	46%	94%	3%	3%	64.6	2.0	13.7
319	32%	0%	0%	33%	99%	0%	0%	718.7	3.2	15.6
403	20%	0%	0%	20%	94%	6%	0%	95.4	6.7	7.8
428	8%	4%	1%	12%	57%	35%	7%	15.0	22.1	4.6
441	5%	6%	1%	12%	56%	40%	4%	12.8	20.4	7.4
496	20%	2%	0%	22%	91%	9%	0%	24.2	4.6	31.7
539	10%	7%	0%	17%	82%	17%	1%	27.0	12.4	8.9

All transported items moved from old to new nest.

The difference between the mean worker traffic to and from the new nest was about 3800, in general accord with the average colony size at Ant Heaven. However, estimating individual colony size by this method gave results that were in poor agreement with colony size estimated from the area of the old and new nest disc (log worker number = −0.44+1.20*log disc area; mean, old nest— 5300 and new nest—1900, respectively). This is probably because counts were not made continuously, and parts of the moves were not recorded. If the missed fraction varied among colonies, agreement with other size estimates would be poor.

Seeds and charcoal were carried from the old nest to the new, almost never the reverse (the reverse is not shown in the figures). Of the dark workers traveling to the new nest, a mean of 20% carried seeds, only 2% carried brood and 1% charcoal. Thus an average of 22% (s.d. = 9.7%) of the dark workers going to the new nest were carrying a load. Eighty percent of these loads were seeds (s.d. = 15%), only 13% were brood (s.d. = 12%), and 7% were charcoal (s.d. = 12%). The low mean rate of brood transport was probably the result of brood being carried in short, intense bursts that the sampling regime often missed. Another way to understand these patterns is as the number of trips required to transport each item. For seeds, this averaged 10.6 (s.d. = 10.4), for brood it averaged 39 (s.d. = 29; one outlier deleted) and for charcoal 10.4 (s.d. = 7.9). Clearly, most workers make most trips back and forth empty-handed.

## Discussion

The high frequency and conspicuousness of nest relocation make the Florida harvester ant an excellent subject for the study of this remarkable phenomenon, and perhaps a model for relocation in other species. Once the relocation has begun, the ants excavate a new nest similar in size and architecture to the original, and simultaneously, through tens of thousands of round trips, gradually move the colony and its contents into the gradually growing new nest. Within this new nest, in spite of the scrambling associated with thousands of burdened and unburdened trips back and forth, they recreate the same vertical organization as in the old nest, with young workers and brood in the deeper zones, seed chambers 50 to 100 cm below ground, and foragers only in the top 15 cm [29]. Moreover, the ants accomplish all this in 4 to 8 days, excavating and moving the equivalent in sand of 1,000 times the weight of all the workers in the colony, and in the process, creating a species-typical nest of striking beauty [25].

The architecture of *P. badius* is characterized by the shapes, sizes and spacing of chambers, as well as by the vertical distribution of percent of the total area [25]. Such vertical organization, both of the ants and of the nest architecture, requires that the ants have information about the depth at which they find themselves. Tschinkel [25] suggested that such information is contained in the gradient of soil carbon dioxide concentration, but he later showed through experiments [26] that the ants do not use these gradients for these purposes. The source from which the ants derive depth information remains unknown.

The present study confirms the general findings in South Carolina with respect to the frequency and distance of relocations [10], [23], [29]. These authors reported nests to move an average of 1.3 to 3.6 m, in the same range as my findings of about 4.1 m. Whether or not these differences are meaningful, it is clear that colonies in both regions do not move far. Carlson and Gentry [23] and Harrison and Gentry [10] noted that colonies moved along their foraging trails but did not report the compass heading of moves. However, their figures show no distinct compass direction of relocations, suggesting randomness as in the present study. Whereas the Ant Heaven population completed moves in 4 to 8 days, Carlson and Gentry [23] reported 1 to 2 weeks, distinctly longer. Sixty to 90% of colonies in South Carolina moved once a summer, with 6 to 41% moving multiple times. Whether this is different from the Ant Heaven population cannot be judged with confidence because inter-annual variation clearly exists, and sample sizes were very different. The localization of foragers to the top few cm of the nest was also established with P^32^-marked foragers by Golley and Gentry [22], as was a mean colony size of 4 to 6 thousand workers.

A regional difference was that in South Carolina (34.34°N latitude) 100% of experimentally-shaded colonies moved, compared to 18% of the unshaded controls. The Florida colonies (30.36°N. latitude) did not respond to shading (unpubl. observ.), perhaps because soil temperatures were higher than in South Carolina where shading reduced temperatures down to a depth of 30 cm [23]. Shading has also been tested on *Polyrhachis ammon*, which emigrated in response to shading [30] and *Ectatomma ruidum*, which sought shade [31]. Intercolony spacing is often postulated to be the cause of relocation, or at least to increase spacing, but empirical data rarely show this to be the case [32], [12]. Spacing did not change significantly in the South Carolina population of *P. badius* as a result of relocations [10]. In the Ant Heaven population, moves were not related to neighborhood densities, nor did the direction of movement bear any relationship to closest neighbors. This is in contrast to nests of *Blepharidatta* spp. whose frequent moves increased their distance from neighbors [33], and whose direction of movement was usually away from neighbors. In two species of slavemaking ants, relocation shortened the subsequent raiding distance [34]. Relocation near food plants has also been proposed [35], but this factor is probably more closely associated with polydomous species [36]–[38]. Ultimately, for *P. badius*, as for most ground-nesting species that practice intrinsic relocation, the factors that trigger the move are unknown. That flooding of its subterranean nest induced relocation in *Atta columbica* seems reasonable [5]. In cavity-nesters, it is not surprising that experimental destruction of the cavity nest triggers emigration [10], [13] as does its invasion by natural enemies, including other ants [17], [18], [39].

Relocation is strongly seasonal and is related to seasonal foraging patterns. Colonies do not forage during the Florida “winter”, from early November until early March, and rarely appear on the surface during this time. Relocation should be seen in this context and in relation to weather patterns. In north Florida, April and May are usually dry months, with May receiving 10 cm of rain in 2012 and 4 cm in 2013. The weather warms rapidly during May so that by the end of the month, the daily highs are more or less similar until mid-September. Surface temperatures are unlikely to be the sole limitation to relocation — relocations continued during October and November when the temperatures were lower than in May. It is possible that the difference in relocation rates between the first half and the second half of the year resides in the different nature of the forager population. Once foraging begins in March, the proportion of the colony engaged in foraging builds to a maximum in July, then declines until it ceases in November [28]. The workers that forage until early July eclosed after September in the previous year and are much longer-lived and older at the onset of foraging than the short-lived cohort that follows and forages until November [28]. Given the extreme difference in life-span of these two cohorts (>300 days vs. 40 days), and the associated differences in physiology, it is possible that the tendency to relocate is differentially expressed in them.

Because the rate of relocation is so strongly seasonal, estimation of colony residence time or half-life fails to tell the full story. Reported residence times often apply only to the active season, for example, two cavity-nesting *Leptothorax* species had residence times of 16 to 28 days during the summer [19]. Other examples of warm season estimates include 16 or 17 days for *Pristomyrmex pungens* [35], a month to over a year for the Argentine ant [36] and 133 days for *Tapinoma sessile* [40]. Reports of percent moving per year are more readily compared and include 80% for *P. badius* colonies [10], 10% for *P. barbatus* [12] and 80% for *Messor andrei* [32]. Ten percent of *Megaponera foetens* moved per month [41]. In the present study of *P. badius*, the probability of moving was 0.72 in 2012 and 1.15 in 2013, comparable to the rates reported by Harrison and Gentry (1981) [10].

For ground-nesting ants such as *P. badius*, the time required to complete a move is obviously greater than it is for cavity nesters who simply move into an acceptable space. Cavity nesters often complete a move in a few minutes to hours [20], [17], [14]. Soil nesting ants must excavate a nest before or during a move, and this takes much longer, up to a week in *Atta colombica* [5]. Mikheyev and Tschinkel [42] estimated that if all workers of *Formica pallidefulva* participated, it would take about a week to excavate a nest, but no actual moves were observed. A large colony of *P. badius* must excavate up to 5 or 6 l of sand, weighing up to 20 kg with a worker force that never exceeds 10,000.

In several species, relocations are organized and stimulated by an active minority of the ants, usually foragers [14], [20]. This is true even for honeybees [43]. In a sense, how could it be otherwise, as the foragers are the only group that has experience outside the nest? Preliminary marking experiments (unpublished data, methods in [28]) indicated that in *P. badius*, foragers are also the initiators of nest relocations. The complete cessation of foraging during relocations (C. Kwapich, pers. comm.) suggests that they are engaged in the move throughout its duration. Mark-recapture of workers bringing pellets of sand to the surface of new nests also suggested that these are largely foragers— these marked workers composed a large fraction of foragers after the move was complete, and upon excavation of the new nest, they were located within 20 cm of the surface (unpublished data). As the nests are far deeper than 20 cm, this suggests that excavated sand pellets are moved to the surface in a chain of transport, much as leaf fragments are transported in leaf-cutting ants [44]. Indeed Rink et al. [45] established a *P. badius* colony in the field in layers of colored sand and showed that a good deal of the sand excavated from depth was likely to be deposited within 30 cm of the surface, from which location it was available for transport to the surface. Careful excavation of *P. badius* nests often reveals intact sand pellets strewn on chamber floors (pers. observations). Similar upward and downward subsurface deposition of soil was found in laboratory colonies of *P. occidentalis* [46]. In spite of these hints, the exact role of foragers in nest excavation remains to be uncovered.

How is the new nest site chosen? The relocation decision seems to come in two parts, first, a decision to move is made (probably within the forager group, but based on unknown cues), second, a small group of workers chooses a site and begins excavating a nest, establishing a (usually) weak trail from the old nest. In several cases, two or even three groups of workers initiated small nests in different locations, but the colonies invariably settled on only one of those (pers. obs.). In three cases, colonies initiated a relocation, but abandoned it after two or three days so that no move took place. On two occasions, colonies completed a move, only to return to the old nest in a few days, emphasizing the complexity of this phenomenon. Whether foragers make these decisions, then motivate and guide other workers to join the move is a question for the future.

The same behaviors may underpin all types of nest relocation in both cavity and soil-nesters, including emergency moves, unstable/intrinsic moves, colony fission, and seasonal polydomy in which ant colonies expand into multiple nests during the foraging season and contract back to one or a few nests in the dormant season (for example [40], [36], [37]. All involve similar basic tasks of orientation and transport, as well as responsiveness to social cues that initiate and organize the moves. In the course of evolution, these same behaviors, with some modification, have come to serve multiple kinds of relocation.

## Supporting Information

Table S1 **The complete basic data on which this study was based, including timed counts for all colonies and all moved items, dates, trail temperatures and elapsed times.**
(XLS)
